# Optimising child outcomes from parenting interventions: fathers’ experiences, preferences and barriers to participation

**DOI:** 10.1186/s12889-017-4426-1

**Published:** 2017-06-07

**Authors:** Lucy A. Tully, Patrycja J. Piotrowska, Daniel A. J. Collins, Kathleen S. Mairet, Nicola Black, Eva R. Kimonis, David J. Hawes, Caroline Moul, Rhoshel K. Lenroot, Paul J. Frick, Vicki Anderson, Mark R. Dadds

**Affiliations:** 1 School of Psychology, University of Sydney, Sydney, Australia; 2 School of Psychology, University of New South Wales, Sydney, Australia; 3 School of Psychiatry, Faculty of Medicine, University of New South Wales, Sydney, Australia; 4 Learning Sciences Institute of Australia, Australian Catholic University, Brisbane, Australia, & Department of Psychology, Louisiana State University, Baton Rouge, USA; 5 Royal Children’s Hospital, Murdoch Children’s Research Institute, Departments of Psychology & Paediatrics, University of Melbourne, Melbourne, Australia

**Keywords:** Externalising disorders, Parent–child relationships, Intervention research, Parenting

## Abstract

**Background:**

Early childhood interventions can have both immediate and long-term positive effects on cognitive, behavioural, health and education outcomes. Fathers are underrepresented in interventions focusing on the well-being of children. However, father participation may be critical for intervention effectiveness, especially for parenting interventions for child externalising problems. To date, there has been very little research conducted to understand the low rates of father participation and to facilitate the development of interventions to meet the needs of fathers. This study examined fathers’ experiences of, and preferences for, parenting interventions as well as perceptions of barriers to participation. It also examined how these factors were associated with child externalising behaviour problems, and explored the predictors of participation in parenting interventions.

**Methods:**

A community sample of 1001 fathers of children aged 2–16 years completed an online survey about experiences with parenting interventions, perceived barriers to participation, the importance of different factors in their decision to attend, and preferred content and delivery methods. They also completed ratings of their child’s behaviour using the Strengths and Difficulties Questionnaire.

**Results:**

Overall, 15% of fathers had participated in a parenting intervention or treatment for child behaviour, with significantly higher rates of participation for fathers of children with high versus low levels of externalising problems. Fathers rated understanding what is involved in the program and knowing that the facilitator is trained as the two most important factors in their decision to participate. There were several barriers to participation that fathers of children with high-level externalising problems were more likely to endorse, across practical barriers and help-seeking attitudes, compared to fathers of children with low-level externalising problems. Almost two-thirds of fathers of children with high-level externalising behaviour had not participated in a parenting intervention or treatment. The only significant predictors of intervention participation were severity of child externalising behaviour problems and child age.

**Conclusions:**

The findings have important implications for services seeking to increase father engagement and highlight a number of strategies to enhance the promotion and delivery of parenting interventions to fathers. These strategies include more public health messaging about parenting programs and the importance of father participation.

## Background

The seeds of lifetime achievement, as well as social, physical and mental health are sown early in childhood [1]. Many adult physical and mental health problems have their origins in childhood [2, 3], and evidence suggests that early childhood interventions can have immediate and long-term positive effects on cognitive, behavioural, health and education outcomes [4]. Crucially, however, fathers are largely missing from interventions focussing on the well-being of children. Numerous reviews have highlighted the underrepresentation of fathers in parenting interventions [5], child welfare services [6–8], pediatrics [9, 10], as well as interventions targeting: childhood autism [11]; externalising problems such as oppositional behaviour, temper tantrums and aggression [12]; Attention Deficit Hyperactivity Disorder (ADHD) [13]; and internalising problems such as anxiety [14]. The low level of father engagement is concerning, especially as there is evidence to suggest that father involvement in interventions can lead to improved outcomes for children [15]. There has been very little research conducted with fathers about their needs and preferences for interventions. Such research is critical for developing father-inclusive interventions which may increase father engagement and intervention efficacy, especially among the most at-risk families. The current paper reports on the findings of a survey of fathers regarding their needs and preferences for parenting interventions, and their perceptions of barriers to participation. In this paper, we focus on parenting interventions for childhood externalising problems, as this is the intervention for which most is known in relation to father involvement.

There is significant evidence that parenting interventions, which focus on enhancing the quality and consistency of parenting, produce reliable improvement in externalising behaviour problems [16], with effects lasting up to 20 years after the intervention [17]. The majority of participants in parenting interventions are mothers and many studies do not even report on rates of father participation [5, 18, 19]. When rates have been reported, only around 13–21% of attendees are fathers [13, 20, 21]. Importantly, there is evidence that including fathers in parenting interventions leads to reductions in child externalising behaviours. Lundahl et al. (2008) conducted a meta-analytic review (*k* = 26) and found that father engagement in parenting interventions was associated with reduced child externalising behaviour and improved parenting behaviour in the short-term, but not in the longer-term. However, other research has found long-term improvements in outcomes for children when fathers are included in interventions [22, 23]. It is not surprising that father engagement improves the intervention effectiveness, as inclusion of fathers (and the core parenting team) is likely to be necessary for: (1) addressing father-specific (in addition to mother-specific) risk factors (e.g., harsh, coercive parenting) that may cause or maintain child externalising problems, (2) enhancing inter-parental consistency in implementation of parenting strategies, and (3) reducing parenting conflict, which is in itself a key risk factor for child externalising problems [24].

While interventions appear to be more effective if fathers take part, there is also evidence to suggest that fathers who participate receive fewer benefits from parenting interventions than do mothers. Meta-analyses have demonstrated smaller effect sizes for changes in fathers’ ratings of parenting and child behaviour compared with mothers’ ratings [18, 25], although it should be noted that many studies do not report on outcome measures separately for mothers and fathers [5, 12]. Due to the lack of research, the reasons for the smaller effects for fathers versus mothers are largely unknown. One possibility is that, as parenting interventions have largely been developed for, and empirically tested with mothers, they may not adequately meet the needs of fathers [26]. Therefore, research is required to better understand fathers’ needs and preferences regarding parenting programs.

Little empirical research has investigated reasons for the low rates of father engagement, with available studies characterised by either small samples and qualitative methodologies [27–29], or practitioner surveys [30–32]. Overall, this research suggests that there are likely to be a range of interrelated factors that act as barriers to father engagement, including: (1) *practical factors,* such as fathers’ work commitments and availability of child care; (2) *program factors,* such as content not being relevant for fathers; (3) *personal factors,* such as fathers’ beliefs about help-seeking or awareness of parenting interventions; (4) *family factors,* such as mothers’ facilitation of father engagement, known as ‘maternal gatekeeping’ [31]; (5) *practitioner factors,* such as skills and confidence in engaging fathers, and; (6) *organisational factors,* such as offering sessions outside working hours, and policies and practices regarding father inclusion.

To date there have been only two surveys of fathers to examine their experiences of parenting or participation in parenting programs. The first surveyed 933 Australian fathers [33] and found 11% of fathers reported that they had participated in a parenting program in the last 12 months (a further 6% had consulted a professional about their child’s behaviour). The second surveyed 161 New Zealand fathers [34] and found a lifetime participation rate of 3%. The features of parenting interventions that fathers rated as most important to them were demonstrated program effectiveness, the personal relevance of the program content, and having a trained practitioner run the program [34]. Fathers also preferred less intensive delivery formats involving seminars, father-only groups, television series and internet-based programs, rather than weekly or more intensive programs. Finally, the topics fathers rated as most important were building a positive parent–child relationship, increasing children’s confidence and social skills, and positively influencing children’s development [34]. While these studies provide insights into fathers’ general needs and preferences, the specific needs and preferences of fathers of children with high levels of externalising behaviour problems have not been explored. As noted by Frank et al. [34], to achieve the goal of increasing the reach of parenting interventions, information is needed on the needs of many father groups, including those with low and high externalising problems.

Although parenting interventions can be offered as a universal intervention to all parents in order to prevent the onset or escalation of behaviour problems in children, many interventions target parents of children who are already displaying externalising behaviour problems, as these families are thought most likely to benefit. Despite this, research suggests that only a minority of these families participate in evidence-based parenting interventions [33]. Given the high prevalence of child externalising problems [35] and low rates of program participation, it is particularly important to examine whether parents of children with high externalising behaviours have different needs and preferences to those with low externalising problems, in order to design effective and engaging interventions for this population [34]. Aligning parent preferences with the content and delivery of interventions for specific presenting problems (such as externalising problems) may improve engagement and outcomes [36]. However, most of the research on parent preferences to date has been conducted with mothers [37].

It is also important to examine predictors of participation in parenting interventions to help identify the factors associated with participation of fathers. Only one study to date examined variables associated with help-seeking, which was conceptualised as both participation in a parenting program and consulting a professional about their child’s behaviour [33]. This study found that social advantage (e.g., higher education level) was positively associated with fathers' participation in parenting programs, but negatively associated with consulting a professional about their child’s behaviour; severity of child behaviour difficulty was associated with both help-seeking outcomes. However, this study did not use a standardised measure of child behavioural difficulty. In addition, the conclusions in this paper (that fathers in low socioeconomic circumstances seek help for child behaviour, but not for parenting), are tempered by the small number of fathers endorsing help-seeking and the unclear analytical strategy. Further research is needed with a standardised measure of child outcomes to investigate predictors of help-seeking for fathers.

In summary, research on fathers’ needs and preferences regarding parenting interventions is limited. There is also little research on factors associated with father participation in parenting interventions. Finally, it is unknown whether father preferences differ depending on the levels of child externalising behaviour problems. Thus, there is currently little knowledge to draw from to inform the tailoring or development of father-friendly interventions, especially for families at greatest risk. Given the low rates of father engagement and the reduced efficacy for fathers relative to mothers when they do participate in parenting interventions, it is critical to conduct large-scale community surveys to inform the development of engaging and effective father-inclusive interventions, particularly for fathers of children with high levels of externalising problems. To address these issues, the current study provides data from an online survey of Australian fathers of children aged 2 to 16 years that examined their experiences with parenting interventions, perceived barriers to participation, the importance of a range of factors in their decision to attend, and preferred content and delivery methods. The study also examined whether fathers’ needs and preferences differed on the basis of whether or not their child had elevated levels of externalising behaviour problems. Finally, the study examined the predictors of participation in parenting interventions.

## Methods

### Participants and procedure

Participants were fathers/male caregivers aged over 18 who lived in Australia and had a child aged 2 to 16 years. Potential participants were recruited to participate in the survey from a research panel of 450,000 Australian people. The panel provider used online recruitment such as online messaging and email to make initial contact with potential participants. Prior to completing the screening questions to assess inclusion criteria (i.e., male caregiver, aged over 18 years, caregiver of a child aged 2–16 years, and Australian resident), potential participants were told that it was a survey about parenting that would take approximately 20 min to complete. Only after they had completed the screening questions and were deemed eligible were they given the participant information statement and consent form. If they did not meet the eligibility criteria, they were told that they did not meet the requirements but were not given the reason why they were ineligible. These steps minimised the risk of ineligible participants participating in the survey. Digital fingerprinting was used to ensure that the same respondent did not complete the survey more than once from the same device.

The online survey was available for completion during a two-week period in March 2016. The survey was anonymous and took on average 10 min to complete. Participants were required to read an online participant information sheet and provide consent prior to completing the survey. During the recruitment period, a total of 1478 respondents accessed the survey link. Of those, 189 (13.0%) were not eligible after completing the screening questions. The reasons for exclusion included: not having children (*n* = 75, 39.7%), aged under 18 years (*n* = 29; 15.3%), female (*n* = 13, 6.9%), not having the necessary browser requirements to complete the survey (*n* = 4, 2.1%), and not having children in the 2–16 years age range (*n* = 68; 36.0%). Of the 1289 eligible respondents, 208 (16.1%) did not answer any questions and 51 (3.9%) commenced the survey but dropped out prior to completion. Of the 1030 who completed the full survey, 29 were excluded for completing it too quickly (indicating a likely invalid response), giving a final sample size of 1001. Participants were provided a small reimbursement to compensate them for their time. This incentive was in the form of online points which could be redeemed for a gift card chosen from a website once a certain point threshold was reached. The survey was part of a project called *Like Father Like Son,* which seeks to enhance the engagement of fathers in parenting interventions in Australia.

### Measures

The questions included in the survey were determined through a comprehensive review of existing literature on topics such as barriers to participation, aspects of parenting interventions seen as important, preferred delivery formats and content of interest to fathers. In addition, eight clinical psychologists with extensive experience in delivering parenting interventions helped to generate survey questions, which were then pilot tested with 69 fathers. Based on feedback from the pilot test, items were revised for wording clarity before being included in the final survey.

#### Father and child demographic characteristics

Data was collected on fathers’ age, marital and education status, whether English was the main language spoken at home, and number of children. Respondents with more than one child were asked to select a ‘target child’, about whom to answer further questions. The target child was a child within the 2–16 age range whose behaviour they were most concerned about. If they had no concerns about their child/ren’s behaviour, they were asked to select their youngest child within the 2–16 age range. Respondents were then asked the age and gender of their selected target child, how involved they were in this child’s life, and whether they lived with this child full-time, part-time or not at all.

#### Child externalising behaviour

To assess child externalising behaviour, participants completed the Strengths and Difficulties Questionnaire (SDQ) [38] about the target child. The SDQ is a 25 item questionnaire using a three response option format (not true, somewhat true, certainly true). The questionnaire yields a total difficulty score, and five subscales (each consisting of five items): hyperactivity, conduct problems, peer problems, emotional symptoms and prosocial behaviour. In order to compare fathers of children with high and low externalising behaviour, children were divided into two groups based on their scores on the conduct disorder and/or hyperactivity scales. Children with scores of 4 or above on the conduct disorder scale and/or 8 or above for the hyperactivity scale were considered to have externalising behaviours in the high/very high range (high EXT group), while those who scored in the normal range on both these scales were considered as having low levels of externalising behaviour (low EXT group) [39].

#### Previous participation in parenting intervention

Participants were asked whether they had previously participated in a parenting intervention or treatment for child behaviour problems. If they had previously participated, respondents were asked their perceptions of how helpful this intervention had been for their child’s behaviour and for their own parenting, on a five-point scale ranging from extremely helpful (5) to not at all helpful (1). They were also asked how relevant the program was for them as a father, on a five-point scale ranging from extremely relevant (5) to not at all relevant (1).

#### Perceived barriers

Respondents were asked about perceived barriers to their participation in parenting interventions or treatment for child behaviour problems. Fathers were asked why they had not participated in a parenting program before, or, if they had participated before, what would prevent them from participating again in the future. A series of 20 barriers were listed which fathers could endorse. These included practical barriers (e.g., cost of service, work commitments, long waiting lists); lack of knowledge or awareness about parenting programs (e.g., not knowing whether programs are effective); help-seeking attitudes or beliefs (e.g., not feeling like their child’s behaviour is a problem); and other factors (e.g., worry about being judged, cultural/religious factors). These barriers were considered individually, and an overall index of total barriers for each respondent was created by summing the number of barriers they endorsed.

#### Preferred supplementary content

Participants were provided with a list of topics and asked how interested they would be in receiving information about each topic (in addition to receiving information about core parenting strategies). Topics included: bully-proofing your child, co-parenting, problem-solving without aggression, quality time and play, social skills, and healthy body image. Participants rated their interest on a five-point scale from extremely interested (5) through to not at all interested (1).

#### Program factors important to the decision to participate

Participants rated the importance of different program factors in their decision to participate in a parenting program on a five-point scale from extremely important (5) to not at all important (1). These included practical factors (e.g., convenient location, convenient time); knowledge about the program (e.g., understanding what is involved, knowing that the program has been tested and is effective); practitioner characteristics (e.g., knowing that the facilitator is trained) and recruitment method (e.g., receiving a personal recommendation from another father).

#### Preferred delivery formats

Participants were also asked to rate how likely they were to participate in different program delivery formats on a five-point scale ranging from extremely likely (5) to not at all likely (1). Formats included internet-based, mobile application, weekly groups or individual sessions (both parents and father only), one-off session (both parents and father only) and telephone sessions.

### Procedure

The Human Research Ethics Committee at the University of Sydney provided ethics approval for the study (2015/821). Participants read an online information statement and gave informed consent before commencing the survey. As the survey was online, participants did not provide written consent, but indicated their consent to participate by clicking a box acknowledging that they had read the information statement and they agreed to participate. Potential participants could not complete the survey without clicking this box. The questionnaire was anonymous and no identifying information was obtained.

### Statistical analysis

Data were weighted using post-stratification sampling weights to adjust for potential sampling biases. Weights adjusted the data for under- and over-sampling based on father age and geographic region, against 2011 Australian Bureau of Statistics’ Census data on Male Parents of Children aged 2–16 years [40]. All data and analyses conducted and reported were adjusted for these sampling weights, with the exception of drop out analyses (i.e., weights were not available for the participants who dropped out of the survey).

Initially all variables were examined using descriptive statistics. To compare responses from fathers of children in the high EXT versus low EXT groups, a series of chi-square tests were used for dichotomous dependent variables; analysis of covariance (ANCOVAs) and multivariate analysis of covariance (MANCOVAs) were conducted for continuous dependent variables. As there were significant differences between high and low EXT groups on two socio-demographic characteristics (see participants section below), two covariates were included in analyses with continuous outcomes variables. Due to the challenges in controlling for covariates in the chi-square tests, significant results were followed up by stepwise logistic regressions using forward entry (covariates on the first step, EXT group on the second step) to determine whether the significant effect of EXT group across outcomes remained after controlling for covariates.

To investigate predictors of father attendance in parenting programs or treatment for child behavioural problems, a logistic regression analysis was conducted with seven independent variables simultaneously entered into the model: father education, father age, father relationship status, number of children, child age, child gender and level of child externalising behaviour.

## Results

### Participants

To compare fathers who dropped out of the survey (*n* = 51) with those who remained (*n* = 1001), t-tests and chi-square tests were performed across socio-demographic variables. Significant differences emerged for two variables: fathers’ age and number of children. Fathers who dropped out of the survey were significantly older (*M* = 46.3, *SD* = 11.0 years) than completers (*M* = 42.3, *SD* = 9.6 years), *t* (1051) = −2.9, *p* < .01, and had significantly more children (*M* = 2.5, *SD* = 1.5 vs. *M* = 2.1, *SD* = 1.1), *t* (1045) = −2.1, *p* < .05.

The socio-demographic characteristics for the entire sample are displayed in Table 1. Fathers in the final sample were on average 42 years of age (range = 18–80 years), and the majority were married/defacto. Just over half of respondents had completed a university degree, just over one-third had completed secondary school or equivalent, and less than one in ten had completed grade 10 or less. Respondents had on average 2 children (range = 1–11). The target child was male in just under two-thirds of families, and aged 8 years on average. The majority of fathers indicated the target child lived with them full-time and most fathers described themselves as extremely or very involved in child rearing. The majority of fathers also indicated that English was the main language spoken at home.

**Table 1:** Sample Characteristics for fathers in the High and Low Externalising Groups

Variable	Total sample	High EXT (*n* = 296)	Low EXT (*n* = 705)	*t* or χ^2^ value
		Mean (SD)	Mean (SD)	*t*
Father age (years)	42.30 (9.31)	39.11 (9.42)	43.64 (8.93)	7.21***
Number of children	2.09 (1.06)	2.09 (1.27)	2.09 (0.97)	−0.06
Age of target child	8.89 (4.37)	8.67 (4.45)	8.96 (4.34)	0.96
		*n* (%)	*n* (%)	χ^2^
Child gender male	603 (60.2)	199 (67.2)	404 (57.3)	8.57**
English language	920 (92.0)	279 (93.9)	642 (91.1)	2.33
Father education				
University	545 (54.4)	171 (57.8)	373 (53.0)	4.27
Secondary school	378 (37.8)	98 (33.1)	280 (39.8)	
Grade 10 or less	78 (7.8)	27 (9.1)	51 (7.2)	
Married/defacto	867 (86.7)	256 (86.5)	611 (86.8)	0.17
Child living arrangement				
Full-time with father	881 (88.1)	270 (90.9)	611 (86.8)	3.49
Part-time with father	75 (7.5)	16 (5.4)	59 (8.4)	
Not living with father	45 (4.5)	11 (3.7)	34 (4.8)	
Involvement in child rearing				
Extremely involved	433 (43.3)	132 (44.6)	301 (42.8)	6.54
Very involved	420 (41.9)	112 (37.8)	308 (43.8)	
Somewhat involved	127 (12.7)	45 (15.2)	81 (11.5)	
Not very involved	16 (1.6)	4 (1.4)	12 (1.7)	
Not at all involved	5 (0.5)	3 (1.0)	2 (0.3)	

***p < .001, **p < .01, *p < .05; all data and analyses adjusted for sampling weights

Overall, 296 (29.6%) fathers had children who scored in the high EXT group and the remaining 705 (70.4%) had children who scored in the low EXT group. Socio-demographic characteristics for the high and low EXT groups are displayed in Table 1, along with the t-tests and chi-square analyses for comparison between the two groups. Fathers of children in the high EXT group were significantly younger when compared to fathers of children in the low EXT group. In addition, a significantly higher proportion of the children in the high EXT group were boys compared to the low EXT group. These two variables (father age and child gender) were included as covariates in subsequent analyses comparing the high and low externalising groups.

### Previous participation in parenting interventions

Of the total sample, 153 (15.3%) fathers indicated they had previously participated in a parenting program or treatment for child behaviour problems. Those who had participated rated the intervention an average of 3.66 (*SD* = 1.00) out of 5 for helpfulness for child behaviour and 3.62 (*SD* = 1.00) for helpfulness for parenting. They also rated the intervention an average of 3.71 (*SD* = 0.93) for relevance to them as a father.

Participation rates differed according to the level of child externalising problems. Of the fathers who had participated in a parenting intervention, significantly more had children in the high externalising group (*n* = 88, 57.5%) than in the low externalising group (*n* = 65, 42.5%), χ^2^(1, *N* = 1001) = 67.73, *p* < .001. Thirty percent (88/296) of fathers with a child in the high EXT group indicated that they had participated in a parenting program or treatment for child conduct problems. Univariate ANCOVAs showed that there were no significant differences between the high and low EXT groups in terms of perceived helpfulness of the program for child behaviour [*F* (1, 161) = 0.13, *p* = 0.72] or parenting [*F* (1, 161) = 0.34, *p* = 0.56], or relevance of the program to them as fathers [*F* (1, 167) = 0.54, *p* = 0.47], after controlling for covariates.

### Perceived barriers to participation

Table 2 displays the number and proportion of fathers endorsing a range of barriers to participation in parenting programs and comparison of the high and low EXT group for each barrier. The most endorsed barriers for the entire sample (after ‘I don’t feel like my child’s behaviour is a problem’ and ‘I don’t feel like I need help with my parenting’) were: cost of service (20%), work commitments (20%), not knowing whether the program is effective (17%), not knowing what the program is about (16%), and not being aware of parenting programs (16%).

**Table 2:** Proportion of Fathers Experiencing Barriers to Treatment by Level of Child Externalising Disorders

	Total		High EXT		Low EXT		
Barrier	*n*	(%)	*n*	(%)	*n*	(%)	χ^2^
*Practical*							
Cost of service	196	(19.6)	80	(26.9)	117	(16.6)	14.15 *
Work commitments	196	(19.6)	78	(26.3)	118	(16.8)	11.98
No time to participate	149	(14.9)	50	(16.9)	99	(14.0)	1.34
Programs/services not at convenient location	87	(8.7)	36	(12.1)	51	(7.2)	6.30
Programs/services not at convenient time	84	(8.4)	34	(11.5)	49	(7.0)	5.61
Long waiting lists	69	(6.9)	40	(13.5)	29	(4.1)	28.52 *
Problems with transport	43	(4.3)	28	(9.5)	15	(2.1)	27.26 *
No child care	39	(3.9)	24	(8.1)	14	(2.0)	21.35 *
*Knowledge*							
Not knowing whether the programs are effective	172	(17.2)	58	(19.5)	114	(16.2)	1.66
Not knowing what the program is about	161	(16.1)	52	(17.6)	108	(15.3)	0.77
I didn’t know about parenting programs before	160	(16.0)	46	(15.5)	114	(16.2)	0.07
I don’t know where to go to participate in a parenting program	149	(14.9)	39	(13.2)	110	(15.6)	0.99
*Attitudes and Beliefs*							
I don’t feel like my child’s behaviour is a problem	396	(39.6)	51	(17.2)	345	(48.9)	88.21 *
I don’t feel like I need help with my parenting	288	(28.8)	47	(15.8)	242	(34.3)	34.85 *
I think my child’s problems require treatment of the child, not the parent	90	(9.0%)	48	(16.2)	42	(6.0)	26.81 *
I don’t think programs are suitable for fathers	55	(5.5)	29	(9.8)	26	(3.7)	14.67 *
I feel that it’s a mother’s role to parent the children	30	(3.0)	13	(4.4)	17	(2.4)	2.78
*Other*							
Worry about being judged	81	(8.1)	43	(14.5)	38	(5.4)	23.40 *
I don’t feel comfortable asking for/receiving help with parenting or child issues	72	(7.2)	26	(8.8)	45	(6.4)	1.81
Previous negative experience with mental health professionals	38	(3.8)	19	(6.4)	20	(2.8)	7.08
Cultural/religious factors	32	(3.2)	23	(7.7)	10	(1.4)	26.25 *
My partner attended without me and didn’t encourage me to participate	29	(2.9)	19	(6.4)	10	(1.4)	18.39 *

*High EXT* fathers of children with high or very high levels of externalising disorders (conduct problems or hyperactivity), as indicated by the Strengths and Difficulties Questionnaire (SDQ), *low EXT* fathers of children with close to average or slightly raised levels of externalising disorders, as indicated by the SDQ; *N* = 1001, *df* = 1 for all Chi Square comparisons by level of externalising disorders; *Bonferroni-corrected *p* value (*p* < .002); all data and analyses adjusted for sampling weights

Fathers in the high EXT group were significantly more likely than those in the low EXT group to endorse several factors as barriers to participation. These included practical barriers such as cost of the service, long waiting lists, problems with transport and lack of child care (see Table 2). Fathers in the high EXT group were also significantly more likely to endorse the belief that child behaviour problems require treatment of the child rather than the parent, and that programs are not suitable for fathers, compared to fathers in the low EXT group. In addition, worry about being judged, cultural/religious factors, and their partner attending without encouraging their participation were more frequently reported as barriers to participation for high EXT fathers compared to low EXT fathers. On the other hand, low EXT fathers were more likely to endorse that they did not feel like their child’s behaviour was a problem and that they did not feel like they needed help with parenting. Follow-up analyses on these significant chi-square tests demonstrated that the differences between high and low EXT groups remained significant after controlling for the covariates.

When examining the total number of barriers endorsed overall, there was a significant difference between high and low EXT fathers, after controlling for covariates. High EXT fathers endorsed a significantly greater number of barriers overall (*M* = 3.01, *SD* = 2.30) compared to low EXT fathers (*M* = 2.48, *SD* = 2.30), *F* (1, 1090) = 11.76, *p* < .001.

### Preferred supplementary content

Table 3 displays fathers’ ratings of interest in receiving information across six topics for the entire sample, and separately for the high and low EXT groups. For the entire sample, the topics with the highest ratings were ‘bully-proofing your child’, ‘teaching your child social skills’, and ‘encouraging child development through quality time and play’. The MANCOVA for comparison between high and low EXT groups found significant differences in ratings of preferred topics, *F* (6, 1081) = 5.67, *p* < .001, after controlling for covariates. Follow-up univariate ANCOVAs were conducted to examine the significant effects for each topic (see Table 3). The high EXT group was significantly more interested than the low EXT group in receiving information on co-parenting, helping their child solve problems without aggression, and teaching their child social skills.

**Table 3:** Perceived Interest in Different Topics by Level of Child Externalising Disorders

Topic	Total *M* (*SD*)	High EXT *M* (*SD*)	Low EXT *M* (*SD*)	*F*
Bully-proofing your child (how to stop your child from bullying or being bullied)	3.72 (1.08)	3.84 (1.05)	3.68 (1.09)	2.42
Teaching social skills to your child	3.70 (1.04)	3.90 (0.99)	3.63 (1.06)	10.50 *
How to encourage child development through spending quality time and playing with your child	3.61 (1.07)	3.73 (1.06)	3.56 (1.08)	1.64
Encouraging a healthy body image in your child	3.58 (1.06)	3.68 (1.03)	3.55 (1.08)	1.76
How to help your child solve problems without aggression	3.58 (1.11)	3.81 (1.04)	3.49 (1.13)	15.35 *
Co-parenting (how to work with your partner as a team in raising your child)	3.49 (1.12)	3.74 (1.06)	3.40 (1.13)	15.06 *

*High EXT* fathers of children with high or very high levels of externalising disorders (conduct problems or hyperactivity), as indicated by the Strengths and Difficulties Questionnaire (SDQ), *low EXT* fathers of children with close to average or slightly raised levels of externalising disorders, as indicated by the SDQ; *N* = 1001, *df* = 1 for all ANCOVAs comparisons by level of externalising disorders; * Bonferroni-corrected *p* value (*p* < .008); all data and analyses adjusted for sampling weights

### Program factors important to the decision to participate

Table 4 displays the ratings of perceived importance of a range of program factors and results for comparisons between the high and low EXT groups. Overall, most factors received relatively high mean ratings of importance (at least 3.5/5). The exceptions were male facilitator, receiving a personal recommendation from another father and receiving a personal invitation from the facilitator, which received lower ratings. The highest ratings were for understanding what is involved in the program and for knowing the facilitator is trained.

**Table 4:** Perceived Importance of Factors in Determining Participation in Treatment by Level of Child Externalising Disorders

Factor	Total *M* (*SD*)	High EXT *M* (*SD*)	Low EXT *M* (*SD*)	*F*
*Practical*				
The location is convenient	3.87 (0.92)	3.95 (0.88)	3.84 (0.94)	2.41
The program is on at a convenient time	3.88 (0.96)	3.98 (0.90)	3.83 (0.99)	4.10
*Knowledge*				
Understanding what is involved in the program	3.91 (0.92)	3.91 (0.89)	3.92 (0.94)	0.19
Knowing the program has been tested/is effective	3.89 (0.94)	3.96 (0.91)	3.87 (0.96)	0.46
Knowing the program designed for fathers & mothers	3.77 (0.98)	3.84 (0.93)	3.75 (1.01)	0.41
Having information about the likely benefits	3.76 (0.90)	3.83 (0.84)	3.75 (0.92)	0.76
*Practitioner Characteristics*				
Knowing that the facilitator is trained	3.94 (0.97)	3.98 (0.89)	3.87 (0.96)	0.81
Feeling like the facilitator understands me	3.71 (0.96)	3.80 (0.96)	3.67 (0.97)	1.10
Male facilitator	2.76 (1.22)	3.17 (1.22)	2.57 (1.18)	39.59 *
*Exposure Method*				
Getting a recommendation from another father	3.20 (1.12)	3.39 (1.13)	3.10 (1.12)	6.91
Receiving a personal invitation from the facilitator	3.15 (1.14)	3.46 (1.12)	3.01 (1.13)	21.35 *

*High EXT* fathers of children with high or very high levels of externalising disorders (conduct problems or hyperactivity), as indicated by the Strengths and Difficulties Questionnaire (SDQ), *low EXT* fathers of children with close to average or slightly raised levels of externalising disorders, as indicated by the SDQ; *N* = 1001, *df* = 1 for all ANCOVAs comparisons by level of externalising disorders; * Bonferroni-corrected *p* value (*p* < .004); all data and analyses adjusted for sampling weights

The MANCOVA for comparison between high and low EXT groups found significant differences in ratings of perceived importance across all factors listed, *F* (11, 1076) = 5.04, *p* < .001, after controlling for covariates. Univariate ANCOVAs showed high EXT fathers rated having a male facilitator as significantly more important to their decision to participate compared to low EXT fathers (see Table 4). However, for both groups, this factor ranked as least important overall (in terms of average importance ratings). High EXT fathers also rated having a personal invitation from the facilitator as significantly more important to their decision to participate compared to low EXT fathers, but even so, this received a relatively low average importance score.

### Preferred delivery formats

Table 5 displays fathers’ ratings of perceived likelihood of participating in different formats of parenting programs for the entire sample and comparisons between the high EXT and low EXT groups. For the overall sample, an internet-based parenting program was rated highest followed by a brief one-off seminar. The MANCOVA for comparison between high and low EXT groups found significant differences in ratings of perceived likelihood of participation across all formats listed, *F* (9, 1078) = 12.61, *p* < .001, after controlling for covariates. Univariate ANCOVAs showed that across all program formats, high EXT fathers gave significantly higher ratings of likelihood of participation compared to low EXT fathers (see Table 5).

**Table 5:** Perceived Likelihood of Participating in Treatment by Level of Child Externalising Disorders

Factor	Total M (SD)	High EXT M (SD)	Low EXT M (SD)	F
*Technology-Based*				
Internet-based parenting program	3.18 (1.22)	3.51 (1.13)	3.06 (1.22)	20.89 *
A mobile phone app	2.65 (1.29)	3.13 (1.30)	2.44 (1.22)	42.57 *
Phone sessions	2.26 (1.15)	2.82 (1.29)	2.01 (1.01)	91.05 *
*Single Sessions*				
A one-off seminar (both parents)	3.07 (1.16)	3.38 (1.09)	2.95 (1.16)	22.72 *
A one-off seminar (father only)	3.02 (1.15)	3.29 (1.14)	2.90 (1.14)	18.72 *
*Weekly Sessions*				
Group sessions (both parents)	2.82 (1.15)	3.19 (1.08)	2.66 (1.12)	33.16 *
Group sessions (father only)	2.76 (1.13)	3.15 (1.17)	2.60 (1.07)	36.65 *
Individual sessions (both parents)	2.83 (1.18)	3.32 (1.16)	2.63 (1.13)	60.66 *
Individual sessions (father only)	2.77 (1.14)	3.13 (1.17)	2.60 (1.09)	38.06 *

*High EXT* fathers of children with high or very high levels of externalising disorders (conduct problems or hyperactivity), as indicated by the Strengths and Difficulties Questionnaire (SDQ), *low EXT* fathers of children with close to average or slightly raised levels of externalising disorders, as indicated by the SDQ; *N* = 1001, *df* = 2 for all ANCOVAs comparisons by level of externalising disorders; * Bonferroni-corrected *p* value (*p* < .008); all data and analyses adjusted for sampling weights

### Predictors of participation in parenting programs

A logistic regression was conducted to examine factors predicting participation in parenting intervention for the overall sample (see Table 6). The model for the overall sample was significant χ^2^ (7, *N* = 1001) = 84.25, *p* < .001, but only two significant predictors were found. These predictors were severity of child externalising behaviour and age of child, although the latter was only marginally significant. For each additional year of child’s age, fathers were 1.05 times more likely to participate in parenting programs. For every additional point a child was rated on the SDQ externalising scale, fathers were 1.25 times more likely to participate in a parenting program.

**Table 6:** Logistic regression model predicting attendance at parenting program or treatment for child conduct problems for the entire sample

	*B*	*SE*	Odds Ratio	95% CI for Odds Ratio	
				Lower	Upper
Parent Age	0.01	0.01	1.01	0.99	1.04
Relationship status (single)	−0.43	0.25	0.65	0.39	1.07
Education (year 10 or less)	0.19	0.19	1.21	0.84	1.77
Child gender (male)	0.15	0.19	1.16	0.79	1.69
Child age	0.05	0.02	1.05*	1.00	1.10
Number of children	0.09	0.08	1.09	0.93	1.28
SDQ externalising subscale	0.22	0.03	1.25**	1.18	1.31

*SDQ* Strengths and Difficulties Questionnaire; *N* = 1001, * *p* < .05, **p < .001; all data and analyses adjusted for sampling weights

## Discussion

This paper examined fathers’ experiences with parenting interventions, perceived barriers to participation, and preferred intervention content and delivery methods. It also explored whether preferences differed for fathers of children with high versus low levels of externalising problems, and examined predictors of participation in parenting interventions. Overall, 15% of fathers in the current study reported lifetime participation in a parenting program, which is in keeping with previous research that found 16% of Australian fathers had either participated in a parenting intervention in the previous year or consulted a professional about their child’s behaviour in the past 6 months [33], although higher than the 3% lifetime participation rate reported in a survey with New Zealand fathers [34]. In the current study, those who had participated in a parenting intervention or treatment for child behaviour rated the intervention relatively highly in terms of helpfulness for parenting and child behaviour, and relevance to them as fathers. This suggests that programs available in the community are generally perceived as both relevant and helpful to fathers who participate in them.

Rates of participation in parenting programs or treatment for child behaviour were higher among fathers of children with high than low levels of externalising problems, which is consistent with previous research that found that parents (predominantly mothers) are more likely to participate in parenting programs if they report higher levels of child behavioural problems [41–43]. However, it is important to highlight that it is not possible to determine what impact participation in parenting programs had on levels of externalising problems, given that fathers were asked to report on *current* levels of child behaviour and *retrospective* program participation. We also did not ask fathers about the timing of previous program participation. Given that just under two-thirds of fathers who reported prior program participation had a child with clinical levels of externalising problems, it is possible that the program was of limited effectiveness for child behaviour, or alternatively that children’s behaviour improved to some extent, but remained at clinical levels. It is also possible that program participation took place a number of years prior, with the impact on levels of child behaviour diminishing over time. Finally, we did not ask whether the program or treatment received was specifically in relation to the target child for whom the father answered the SDQ, so it is possible that treatment was for another child in the family. While future research should include questions about timing of, and target for, program participation, it does appear that a significant proportion of fathers who reported previous participation in parenting interventions or treatment for child behaviour problems may require further assistance for their child’s behaviour.

Overall just under one in three fathers in the present study rated their child as having conduct problems or ADHD symptoms at levels that were classified as being high or very high. These rates are higher than those reported in a recent Australian prevalence survey [44] where 10.5% of children aged 4–10 years met the criteria for conduct disorder (8.8% for 11–17 years) and 13.0% of children aged 4–10 years met the criteria for hyperactivity (13.8% for 11–17 years). This result may indicate that fathers of children with high levels of externalising behaviour problems were more likely to participate in the survey. Just less than one in three fathers who had a child with high externalising behaviour reported participation in a parenting program or treatment for child behaviour, which is very similar to father participation rates reported in previous research [33], and suggests that many fathers who may be in need of assistance are not receiving help. This finding highlights the need for services and interventions to implement strategies to enhance the engagement of fathers, especially for those fathers who have concerns about their child’s behaviour.

The only significant predictor of participation in parenting programs or treatment for child behaviour problems (aside from child’s age) was severity of child’s externalising behaviour problems, which confirms previous research with fathers [33], and parents generally [45]. Contrary to previous research, however, socio-demographic variables such as education levels did not predict attendance at parenting programs [33, 45]. This is an encouraging finding as it suggests that fathers from a range of socio-demographic backgrounds are seeking help for parenting and child behaviour problems, at least for the current sample of fathers. It is not surprising that the older the child’s age the more likely the father was to have participated in a parenting program, as increased child age would have presented more opportunities to participate over time. It should be noted that in previous research on factors associated with fathers’ help-seeking, participation in a parenting program and seeking professional help for child behaviour were examined separately, and a different pattern of findings emerged for each outcome variable [33]. That is, higher participation in parenting programs was associated with high social advantage and consulting a professional about child behaviour was associated with lower social advantage. However, in the present study we included a help-seeking variable that examined both participation in parenting program and/or treatment for child behaviour problems, given that we expected that most interventions for child behaviour problems would likely involve some parenting strategies. Clearly, further research is needed to examine predictors of parenting program participation for fathers, and to explore whether predictors differ based on the type of program or intervention received.

Internet-based parenting programs and brief parenting programs were the most preferred delivery formats, both for the overall sample and for the high and low externalising groups, which is consistent with previous research with samples of fathers [34] and (predominantly) mothers [46]. It is not surprising that these less intensive interventions are preferred over weekly individual or group programs, as they make fewer demands on families and may also help address barriers such as child care and transportation difficulties. As there is increasing research to demonstrate the effectiveness of internet-based parenting programs [47] and brief parenting programs [48], these program formats should be made widely available in the community. The findings also point to topics of interest to fathers such as bully-proofing your child, teaching social skills to your child and encouraging child development through quality time and play. While these topics differed to those included in previous surveys [34], they highlight potential content that could be added to parenting interventions to make them more appealing for fathers.

This study provides important information about fathers’ perceived barriers to participation. Cost of the service emerged as one of the most frequently rated barriers, which is surprising given many community-level parenting programs are offered free of charge, and may relate to costs in consulting private practitioners such as psychiatrists, psychologists and paediatricians. Other key barriers identified by fathers included work commitments and not knowing whether the program is effective, which were also found to be key barriers in previous research [34], and suggest that program flexibility and informing fathers about the program’s evidence base may be especially important when engaging fathers. Almost one in six fathers indicated that they were not aware of parenting programs at all, or did not know where to go to participate, which points to the need for media campaigns to increase community awareness about the availability and purpose of parenting programs.

Fathers of children in the high externalising group endorsed significantly more barriers overall, across practical barriers, help-seeking attitudes and other factors, compared to the low externalising group. As fathers of children in the high externalising group had higher levels of participation in parenting programs than the low externalising group, it is likely that they had greater awareness of barriers to participation such as long waiting lists, transport difficulties and lack of available childcare. There were also a range of other barriers that were more frequently endorsed by fathers of children in the high externalising group than the low externalising group, including factors related to stigma (worry about being judged), maternal gatekeeping (my partner attended without me and did not encourage my participation), and help-seeking attitudes (problems with my child’s behaviour require treatment of the child, I don’t think programs are suitable for fathers). Stigma has been found to be predictive of lower levels of help-seeking in parents of children with behavioural problems [49] which indicates the importance of normalising attendance at parenting programs, both in promotional materials and by practitioners who are working with families. It is also important for promotional materials to provide information in order to address misconceptions that child behavioural problems require treatment of the child only rather than the parents, and that programs are not suitable for fathers. Given that fathers in the high externalising group were more likely to endorse the barrier that their partner attended without them and did not encourage their participation, it is important for practitioners to emphasise to mothers the importance of engaging fathers, and to develop skills to engage fathers directly as well as through mothers. Together, these findings suggest that it is important for practitioners to be aware that fathers who seek assistance for their child’s externalising problems may experience numerous barriers to help-seeking, and practitioners should be sensitive to fathers’ experiences of these barriers and use collaborative problem-solving when working to address these barriers.

In terms of factors important to their decision to participate in parenting programs, fathers gave highest ratings to: understanding what is involved in the program, knowing the facilitator is trained, knowing the program has been tested in research and is effective, as well as holding the program at a convenient location and convenient time. These findings, which were similar to those of a New Zealand survey [34], suggest that information about what the program involves, training of facilitator and the evidence base for the program should be included in promotional materials, and every effort should be made to hold programs at convenient times and locations for fathers. Preference for a male facilitator received the lowest importance rating of all factors listed. This finding was again consistent with previous research [34], which found male practitioner gender to be the second least important of several factors. While many researchers have recommended increasing the number of male practitioners in order to increase father participation [50], findings from the present study suggest that other factors (such as the training of the facilitator and fathers’ feeling that the facilitator understands them) may be more important to fathers than practitioner gender. However, male practitioner was rated as more important to fathers of children with high than low externalising behaviour, although it was the factor that received the lowest importance rating for the high externalising group. Similarly, fathers of children in the high externalising group were more likely to rate receiving a personal invitation from the facilitator as important, however once again, this factor had one of the lowest average importance ratings across those listed.

The current study has a number of strengths, including a large sample size of community fathers, weighted to ensure representativeness of the sample on father age and geographic region, and the use of a standardised measure of child externalising problems. However, the findings of this study should be interpreted with caution given four key limitations to the study. First, as participants were members of an online panel, they may not have been representative of fathers in the community on factors other than age and location, and this may have introduced bias that resulted in specific findings, such as high preferences for internet-based parenting interventions. The sample also included a preponderance of university-educated fathers from two-parent families, highlighting the possibility that the sample may not be representative of fathers on education and relationship status, and possibly limiting our ability to generalise these findings to high-risk fathers who are most in need of parenting interventions. Second, while steps were taken to ensure only eligible participants took part in the survey (i.e., by not revealing the purpose of the survey prior to completion of screening questions) and that each respondent only completed the survey once (i.e., by using digital fingerprinting), there is no way to confirm that these strategies were successful. Third, it is important to keep in mind that stated preferences of fathers may not necessarily correspond to choices participants make when actually seeking help, as noted by other researchers [46]. Indeed, given that only 15% of fathers had participated in a parenting program and just under one-third said that they did not need help with their parenting, it is likely that many of their responses about barriers and preferences were based on conjecture rather than their actual experiences. Finally, because we did not include a sample of mothers in this study, we cannot contrast whether fathers’ needs and preferences are similar or different to those of mothers. Future research with fathers should aim to include fathers from a range of socio-economic backgrounds, and also aim to compare mothers’ and fathers’ needs and perceptions in relation to parenting interventions.

While further research is needed to replicate and extend these findings with other samples of fathers, this survey is an important first step to understanding fathers’ perceptions of barriers to participation in parenting interventions, their preferences for content and delivery formats. As such, there are several implications of the findings of this study for the promotion and tailoring of current evidence-based parenting programs to enhance father engagement. First, services should provide programs for free or at low cost, hold them at times and locations convenient to fathers and provide a well-trained facilitator. Research has suggested that only around 40% of practitioners report that their service frequently provides sessions outside working hours [51], and this may be critical to increasing father involvement. Second, promotional materials for a program should include the key points which fathers have indicated are important to their decision to attend, such as describing what is involved in the program, that the program has been tested in research and found to be effective, and the level of training of the facilitator. Third, since around one in six fathers indicated that they did not know about parenting programs, more public health messaging is needed about availability and aims of programs in the community and the importance of father participation. Finally, internet-based or brief parenting interventions need to be widely available, as fathers rated these delivery formats highest in terms of their likelihood of participation. Despite these promising findings, it is important to keep in mind that there is a lack of research on whether tailoring programs to meet the needs of fathers (by modifying content or format) results in significantly higher levels of father involvement and/or more effective programs [52]. In addition, caution is needed in adapting interventions to meet the needs and preferences of fathers, as modification to program content could impact on program fidelity and compromise program effectiveness [52].

## Conclusions

This study provides important information about fathers’ needs and preferences in relation to content and delivery of evidence-based parenting interventions. The findings highlight a number of practical, easy-to-implement strategies that can be used to better promote parenting programs and to tailor the delivery of parenting programs to fathers. Only a small number of fathers had participated in a parenting intervention or treatment for child behaviour, and the majority of fathers of children with high levels of externalising behaviour had not received assistance, indicating the need for services and interventions to directly target fathers, especially those with concerns about child behaviour. While this study provides an important first step in addressing the paucity of research on fathers, much more research on the needs and preferences of fathers is needed in order to increase rates of father participation and to maximise the efficacy of parenting interventions in preventing childhood social and health problems, thereby enhancing child outcomes at a population level.

## Abbreviations

ADHD: Attention deficit hyperactivity disorder
EXT: Externalising behaviour
SDQ: Strengths and difficulties questionnaire
